# Recent Advances in Halal Bioactive Materials for Intelligent Food Packaging Indicator

**DOI:** 10.3390/foods12122387

**Published:** 2023-06-16

**Authors:** Farah Ayuni Mohd Hatta, Qurratu Aini Mat Ali, Mohd Izhar Ariff Mohd Kashim, Rashidi Othman, Sahilah Abd Mutalib, Nurul Hafizah Mohd Nor

**Affiliations:** 1 Institute of Islam Hadhari, National University of Malaysia (UKM), Bangi 43600, Selangor, Malaysia; p113728@siswa.ukm.edu.my (Q.A.M.A.); izhar@ukm.edu.my (M.I.A.M.K.);; 2 Research Centre of Shariah, Faculty of Islamic Studies, National University of Malaysia (UKM), Bangi 43600, Selangor, Malaysia; 3 Department of Landscape Architecture, Kulliyyah of Architecture and Environmental Design, International Islamic University Malaysia, Gombak 53100, Kuala Lumpur, Malaysia; rashidi@iium.edu.my; 4 Department of Food Science, Faculty of Science and Technology, National University of Malaysia (UKM), Bangi 43600, Selangor, Malaysia

**Keywords:** active materials, food packaging, bioactive compound, intelligent indicator, halal, food safety and security

## Abstract

Food safety and security are top priorities for consumers and the food industry alike. Despite strict standards and criteria for food production processes, the potential for food-borne diseases due to improper handling and processing is always present. This has led to an urgent need for solutions that can ensure the safety of packaged foods. Therefore, this paper reviews intelligent packaging, which employs non-toxic and environmentally friendly packaging with superior bioactive materials that has emerged as a promising solution. This review was prepared based on several online libraries and databases from 2008 to 2022. By incorporating halal bioactive materials into the packaging system, it becomes possible to interact with the contents and surrounding environment of halal food products, helping preserve them for longer periods. One particularly promising avenue of research is the use of natural colourants as halal bioactive materials. These colourants possess excellent chemical, thermal, and physical stabilities, along with antioxidant and antimicrobial properties, making them ideal candidates for use in intelligent indicators that can detect food blemishes and prevent pathogenic spoilage. However, despite the potential of this technology, further research and development are needed to promote commercial applications and market development. With continued efforts to explore the full potential of natural colourants as halal bioactive materials, we can meet the increasing demand for food safety and security, helping to ensure that consumers have access to high-quality, safe, and nutritious foods.

## 1. Introduction

The food crisis has led to a strong desire to preserve food integrity and safety. According to the World Health Organisation [1], food safety must address the risks of climate change and the outbreak of pests and diseases, such as COVID-19, where 600 million people, or one in every ten, have been affected by food-borne diseases caused by contaminated food. In addition, the persistent weakness in handling and storage infrastructure has led to serious food waste and loss because foodstuffs, especially fresh foods, can be easily exposed to food-borne diseases caused by bacterial, viral, parasitic, fungal, and prion infections due to improper handling and processing. This issue has impacted 10% of the world’s population and results in 420,000 deaths each year [2]. Subsequently, this situation has led to an increase in world hunger and severe food insecurity. Thus, the development of intelligent food packaging incorporating halal bioactive materials is a great solution to the challenges of food safety concerns.

Food packaging was first used in the eighteenth century, and it has been developed and improved since the twentieth century [3] into various types of packaging technologies, including intelligent food packaging technology [4]. Intelligent food packaging technology is an extension and improvement of traditional food packaging, adding sensors and other technologies to extend the shelf life of packaged goods. Intelligent packaging technology is becoming more relevant in response to dynamic shifts in global consumer demand and food industry trends. This alternative offers the potential for preserving food integrity because it supports sustainability as well as food safety and security. Intelligent packaging is intended to be an indicator of environmental conditions in order to detect changes in food conditions such as time–temperature, gas leakage, and level of freshness, which is in line with the current food crisis. Two major components must be considered in the production of good and successful intelligent packaging: packaging material and additives [2,4].

Bio-based packaging refers to biodegradable materials used as food-contact materials. For instance, poly (lactic acid) (PLA) is a green polymer from renewable resources manufactured by the polymerization of lactic acid monomers derived from the fermentation of starch feedstock, which is biodegradable and compostable [5]. PLA has emerged as a substitute for petrochemical-based polymers, which are hazardous to the environment, owing to its easy availability, good biodegradability, and outstanding mechanical qualities. Additionally, biodegradable polymers have a well-grounded role in tackling the waste problem [6]. This polymer also aids in reducing food loss and environmental contamination caused by the excessive dumping of non-biodegradable garbage [3,7].

Bioactive ingredients such as anthocyanin, betalain, carotenoid, and chlorophyll, with superior antioxidant and antimicrobial properties, can be used as additives in food packaging. However, these safe, natural pigments have demonstrated several issues, including low bioavailability and instability under a variety of environmental conditions, including exposure to moisture, oxygen, and light. Encapsulation, including nanoencapsulation and microencapsulation, is an effective method to protect these plant pigments from potentially harmful environmental conditions while allowing targeted and controlled release [8]. For example, these compounds can be encapsulated in the PLA matrix to improve their properties while maintaining nutritional quality by preventing pathogenic spoilage and oxidative deterioration of food products [9,10].

**Figure 1:**
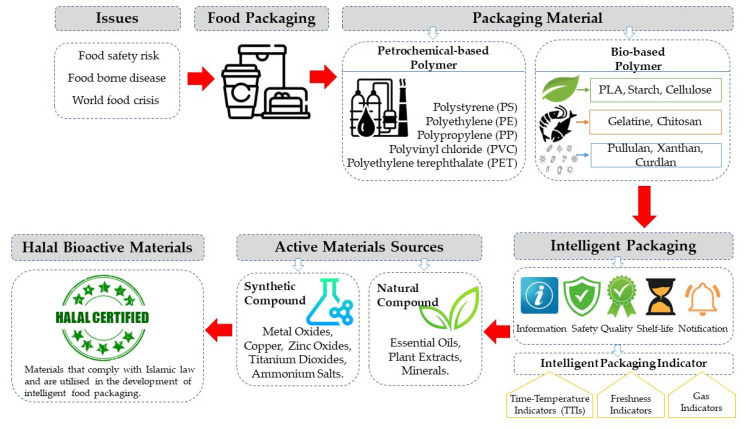
Schematic representation of halal bioactive materials for intelligent packaging indicators addressing food safety concerns.

Halal bioactive materials for intelligent food packaging indicators are materials that adhere to Islamic laws and are used in the development of intelligent food packaging. The use of halal bioactive materials in food packaging helps to provide Muslim consumers with peace of mind when purchasing food products. As halal goes beyond religious reasons to include ethical production, responsible consumption, and environmental protection, it supports the development of sustainable and environmentally friendly packaging solutions that enhance the safety and quality of food products. Figure 1 illustrates the integration of bioactive compounds into the packaging system, highlighting their role in enhancing food safety and mitigating potential risks.

Therefore, this review’s purpose is to discover the recent technological advancements in the application of intelligent food packaging indicators in general and to reveal the potential of halal active compounds with colour-change ability as an intelligent indicator of chemical and physical changes in food products and the environment to sustain their quality and safety. This paper also focuses on halal bioactive materials for intelligent packaging indicators, a topic that has not been covered in the existing literature.

## 2. Materials and Methods

Considering the food safety and security issues that emerged after the COVID-19 pandemic, the recently available resource materials and scientific literature related to food preservation by using food packaging technologies were searched through MDPI, ScienceDirect, Springer Link, Google Scholar, ACS Publications, PubMed, Wiley Online Library, and the databases from international organisation to collect significant information for this review.

The keywords and their combinations used to find relevant articles are “food security”, “food safety”, “food packaging”, “packaging innovation”, “active packaging”, “intelligent packaging”, packaging indicator”, “bioactive compound”, “natural pigment”, “halal bioactive material”, “synthetic pigment”, “natural food additives”, “bio-based polymer”, and “packaging sensors”. The significant documents published from 2008 to 2022 by internationally recognised organisations were considered in this review.

## 3. Packaging for Food Safety and Quality

Food safety is the process of preserving food from risks such as chemical, physical, biological, and radiation hazards; allergens; pests and diseases; legal risks; and commercial frauds that may develop unwanted conditions and threaten the safety and health of end consumers [11,12]. Food safety and qualities are retained in packaging after being produced to ensure further storage, handling, and transportation safety over long distances before arriving to the end consumer. The main objective of food packaging is to keep food secure from oxygen, water vapour, ultraviolet (UV) light, as well as chemical and microbial contamination [13].

Food quality pertains to the attainment of all food-required features that are acceptable to consumers, such as internal and external specifications, recipe and nutritional values, physical appearances, size and weight, flavour, and odour. Petrescu et al. [14] reported that consumer preferences in assessing food quality are based on freshness, taste, and visual appearance. Consumers are also concerned about nutritional facts, ingredients, additives, food origin, packaging, and type of production. Thus, food quality receives considerable attention along with the awareness of improving health, especially after the pandemic [14].

Food packaging is important in the food industry as it ensures that consumers receive high-quality food products. Food packaging acts as food protection from interference or contamination from chemical, physical, and biological sources [15]. It also eases the identification, promotion, and handling of food [16]. Several types of packaging styles are used in the food industry nowadays: bags, cups, pouches, trays, tubes, cans, bottles, and others [3]. Other than protecting food, packaging helps in logistics throughout the transportation, distribution, storage, and retailing procedures. Moreover, it ensures that the food is received in the best condition and is easily handled when it is received by the consumer [17].

### 3.1. Food Packaging Materials

**Figure 2:**
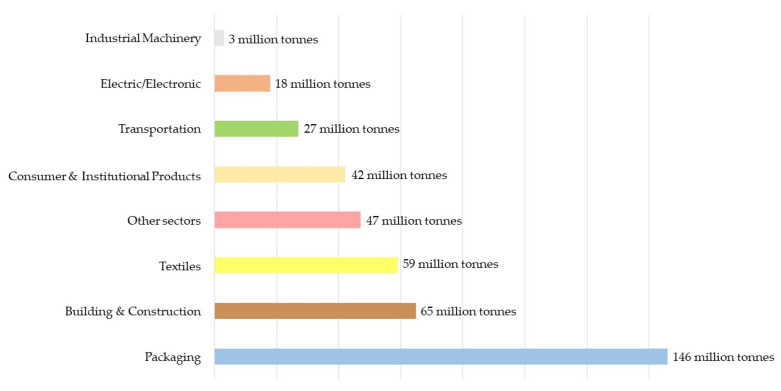
Primary global plastic production by the industrial sector in 2015, measured in tonnes per year, reproduced from [21].

Packaging material for food products comes in several forms and bases including glass, paper, metal, can and others. Plastics, including rigid and flexible types, are the most commonly used materials in the food packaging industry. It is estimated that approximately 36% of all plastics produced are used for food and beverage containers [18,19,20,21]. Figure 2 illustrates the primary global plastic production by various industries, measured in tonnes per year [22]. According to Dominguez et al. [22], recent trends in food packaging materials centre on bio-based or biopolymer materials, which have been used as novel packaging materials, including intelligent packaging. However, the use of petrochemical-based materials remains popular because their production costs are lower than those of bio-based materials. The production of petrochemical- and bio-based plastics from 2020 to 2025 will rise by about 47% and 21%, respectively [23]. Given that conventional plastics are still dominant in the marketplace, however, these engineered-for-immediate-disposal petrochemical-based materials have caused a plethora of environmental challenges [5].

### 3.2. Petrochemical-Based Polymers

The development of various industries led to an extensively increased dependency on petrochemical-based polymers. Petrochemical-based or synthetic polymers, such as polypropylene (PP), polyethylene (PE), polystyrene (PS), polyvinyl chloride (PVC), and polyethylene terephthalate (PET), are derived from petroleum hydrocarbons [18,24]. These polymers are extremely versatile due to the presence of highly desirable properties, such as light weight, flexibility, high resistance, chemical inertness, low cost, and processability. Nonetheless, the usage of fossil fuel-derived and non-renewable plastic materials has increased environmental issues and affected the health of humans. Muncke [19] reported that plastic food packaging has a high possibility of transferring chemicals into packed food. This chemical migration depends on several factors, such as environmental temperature, food types, and the storage period, which may have a negative impact on consumer health. On the other hand, the continuous processing, production, and disposal of these materials have also increased the carbon footprint. Thus, for carbon footprint reduction, ‘fossil carbon’ content must be decreased or replaced with ‘renewable carbon’ content. The substitution will aid in attaining a low carbon footprint, a sustainable environment, effective resource management, and the preservation of human health [23,24].

### 3.3. Bio-Based and Biodegradable Polymers

**Figure 3:**
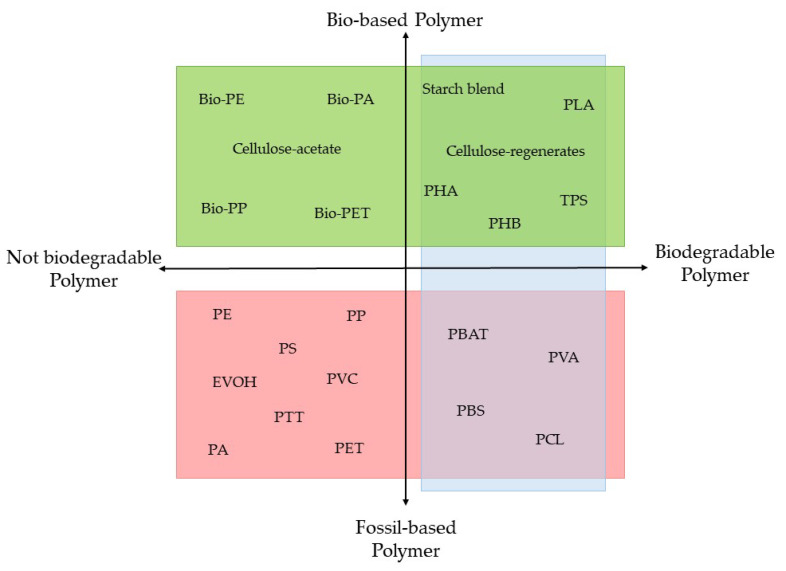
Material coordinate system of bio-based (green) and fossil-based polymers (pink), biodegradable polymer (blue); PE: polyethylene; PA: polyamide; PP: polypropylene; PET: poly(ethylene terephthalate); PLA: polylactic acid; PHA: polyhydroxyalkanoate; PHB: polyhydroxy butyrate; TPS: thermoplastic starch; EVOH: ethylene vinyl alcohol; PVC: polyvinyl chloride; PS: polystyrene; PTT: poly trimethylene terephthalate; PBAT: polybutylene adipate terephthalate; PVA: polyvinyl alcohol; PBS: polybutylene succinate; PCL: polycaprolactone, reproduced from [26].

Bio-based polymers are derived from renewable sources such as starch, cellulose, chitosan, gelatine, poly (lactic acid), polyhydroxybutyrate, polycaprolactone, and polybutylene succinate [25], while biodegradable polymers are either derived from renewable sources or from fossil-based or petrochemical-based polymers [26]. Thus, these two polymer groups have differences in which biodegradable polymers will be completely degraded when exposed to aerobic and anaerobic processes, microorganisms, and water, whereas bio-based polymers can be either biodegradable or not biodegradable [27]. Figure 3 demonstrates the bio-based polymer and the fossil-based polymer to differentiate these polymers. Concerning the serious environmental contamination caused by non-biodegradable plastics, bio-based polymers have recently aroused the scientific community’s interest. This is due to the fact that bio-based polymers have a number of advantages over synthetic ones, including being biocompatible and being less expensive [28]. These advantages include being non-toxic, biodegradable, highly attainable, air permeable, and capable of sealing at low temperatures. [28]. The study of these polymers, which has been continuous since the 1970s, revealed that the most encouraging bio-based polymers are PLA and polyhydroxyalkanoates [29].

Currently, further research is being conducted on PLA to substitute petrochemical-based polymers, especially in the food industry [5]. PLA is one of the most attractive biodegradable polymers for food packaging materials because of its ease of readiness, high biodegradability, and outstanding mechanical characteristics [6]. In addition, gelatine is a well-known bio-based polymer frequently used in food and pharmaceutical industries because it is palatable, soluble, flexible, inexpensive, and has valuable qualities, such as resistance to gas flow [30]. Gelatine, however, can come from both halal and non-halal sources. Gelatine derived from fish and cattle bones, known as halal gelatine, is now widely used in the food and pharmaceutical industries [30]. The other intriguing bio-based polymers, such as pullulan, xanthan, and curdlan, are produced by bacteria and fungi. Pullulan, a bio-based polymer produced by the *Aureobasidium pullulans* strain, stands out above the others due to its unique characteristics and beneficial properties. It has significant potential for use in the packaging industry as a non-toxic, water-soluble polymer with high film formability to build flexible fine layers with specific antibacterial action [31,32]. Moreover, the development of bio-based and biodegradable packaging using wastes such as fish skin and used coffee grounds has previously been studied. The physical and chemical properties of the packaging were investigated, and it was discovered that the coffee extract impregnation improved the packaging’s hydrophobicity, transparency, antioxidant, and antimicrobial activities. The study found potential for using waste to create active packaging in the food and related industries as a sustainable and environmentally friendly substitute for conventional plastic packaging [33].

## 4. Intelligent Packaging

Intelligent packaging is defined as a technology that can communicate and facilitate decision-making to deliver information, enhance safety, assure quality, expand shelf-life, and provide notification of any complication by monitoring alterations in the external and internal environment of packages [13,34]. It aims to observe the product and convey information to consumers, such as the content of a package, the manufacturing date, and storage conditions. The indicator for this intelligent packaging is generally a small item placed either inside or attached to the outside of the package [4]. This information can be used to improve supply chain management, prevent food waste, and enhance the consumer experience.

### Intelligent Packaging Indicator

The function of indicators is to measure and provide a signal for any reaction. Intelligent packaging indicators regulate the presence and absence of any constituent within the range of their concentration and reaction towards foodstuffs. The indicator is projected by visual changes, such as the modification of colour intensities [4]. Three main indicators are used: time–temperature indicators (TTIs), freshness indicators, and gas indicators.

Temperature is one of the factors affecting the shelf life of foodstuffs. The alteration in temperature over time can lead to the growth of microorganisms, which may eventually cause the food to deteriorate and become unsafe for consumption. TTIs may also notify of changes in foodstuffs caused by enzymes, mechanical, chemical, or electrochemical factors. Thus, in the use of TTIs, the temperature of food products must be maintained during handling and transportation before being received by the end consumers [4].

Following that, the freshness indicator performs a similar function to TTIs, but it aids in directly notifying the quality of food products. The freshness indicator will react when metabolites are formed by the growth of microbes in the foodstuff. It will show irreparable visual changes upon the increase in bacteria in the products. Given that this indicator is dependent on metabolites, factors such as the nature of packed foodstuff, flora spoilage, and packaging variety must be considered because they are related to the establishment of metabolites [35].

Gas indicators also ensure food safety and quality. Foodstuff, especially fresh food, starts to decay or is spoiled by microbial fermentation after being packed because of the production and multiplication of carbon dioxide in the packaging. The gas changes with storage time and temperature, which is determined by the type of food, respiratory characteristics, packaging material, and product headspace. Thus, gas indicators may help in tracking the gas level in the headspace of packages to determine the quality of foodstuff [12].

Some intelligent packaging also incorporates active packaging features, such as indicators that can detect the presence of microorganisms or changes in the chemical composition of the food product. Additionally, packaging that incorporates colour changes of active materials indicates the status of the food product, such as changes in temperature, humidity, or other environmental factors, and provides information about the quality and safety of the food product [36,37]. These colour-changing active packaging systems can be considered intelligent packaging because they have the added capability of providing information to the consumer in addition to their functional benefits.

The accuracy of intelligent packaging indicators can vary depending on several factors, including the technology used, the type of information being monitored, and the environment in which the system is used [38]. It is also important to realise that, while this technology can indicate food quality, it is not a guarantee of food safety and should be used in conjunction with other food safety measures, such as proper handling, storage, and preparation [7,39].

## 5. Active Materials for Intelligent Food Packaging Indicator

Active materials play a role in controlling the environment inside the package, increasing the shelf life, and preserving the quality and visual appearance of packed foods. Interestingly, reviews on food packaging with these active materials as additives are abundant [40,41]. Two groups of sources are used as additives: synthetic and natural active compounds. Synthetic compounds, such as metal ions, metal oxides, copper, zinc oxides, titanium dioxides, ammonium salts, and ethylenediaminetetraacetic acid, have been widely used as additives for thousands of years. When used in low concentrations, they have been shown to be the most effective in terms of function and to have the least systemic toxicity to humans. Regardless, controversies and contradictory findings regarding the long-term safety of these synthetic additives to human health must be addressed [41,42]. 

Natural or bioactive compounds with specific functional properties, such as essential oils, plant extracts, and minerals, have all been studied for use in active packaging [43,44]. The appropriate bioactive compound is chosen based on a variety of factors, including its efficacy, compatibility with the food product, and intended end-use application. Therefore, bioactive compounds, especially those from plant sources, are being introduced as alternatives, given that plants consist of pigments, such as chlorophyll, carotenoid, anthocyanin, and betalain. These natural pigments can also be derived from fruits and vegetable wastes and by-products, such as seeds, peels, skins, and leaves [9]. These natural pigments contain a variety of bioactive compounds with physiological effects, such as antioxidants and antimicrobials, and can protect tissues and cells from free radicals and singlet oxygen damage; they can also extend the shelf life of fresh or processed food [10,45,46]. Table 1 exhibits the synthetic and natural active compounds that are currently being used as additives for intelligent food packaging indicators.

There have been a number of studies on the incorporation of active materials into a packaging or coating matrix in order to improve stability and serve as an intelligent indicator. For example, a previous study reviewed the use of anthocyanin-rich extracts from red cabbage as active ingredients in smart bio-based food packaging systems and sensors [65]. The study found that red cabbage anthocyanins act as colourimetric pH-responsive agents that enable reliable real-time monitoring of the qualitative qualities of packaged food products and help maintain the shelf life by inhibiting microbial growth and oxidative deterioration. Another study reviewed the current developments in the production of intelligent, active, and bioactive biopolymer-based films containing betalains [49]. The study discovered that betalains are adaptable natural pigments with a variety of bioactivities, making them ideal colourimetric indicators due to their ability to detect changes in food pH as well as their antibacterial and antioxidant properties.

These studies show that bioactive materials could be added to a packaging or coating matrix to make it more stable and act as an intelligent indicator of the quality and safety of food. It is important to note, however, that the specific properties and effectiveness may vary depending on a variety of factors, including the specific active ingredients used, the type of packaging matrix, internal gas composition control, and the specific food product being packaged [66]. The use of bioactive materials in intelligent food packaging should be carefully designed to ensure the stability and effectiveness of the bioactive compounds. For instance, the incorporation of these compounds into a PLA matrix can help to protect them from degradation and oxidation [67].

### Halal Bioactive Materials for Intelligent Packaging

Polymers are typically made from petroleum-based raw materials. Because of the global problem of pollution, alternative eco-friendly and biodegradable polymers are in high demand. Biopolymers are classified into three types based on their origin and method of production: directly extracted from biomass, synthesised bioderived monomers, and microorganisms [68]. Animal-based and plant-based materials are both viable options for producing edible films and intelligent packaging. The use of either type of material depends on factors such as the intended application, the desired properties of the packaging, and the sustainability and ethical considerations of the material [69]. The primary concern for Halal consumers is the use of additives derived from indefinite sources in the manufacture of polymer resins.

For Muslim consumers, the use of biopolymer materials or additives derived from non-halal sources such as animal blood and protein such as collagen, gelatine, and keratin from the non-halal animal is prohibited. According to MS 2565:2014 Halal Packaging–General Guidelines published by the Department of Islamic Development Malaysia (JAKIM), “halal packaging” refers to packaging materials and containers that comply with Islamic law, which includes not only the ingredients used in the packaging but also the manufacturing process and handling procedures [70]. The guidelines provide a set of criteria that must be met for packaging to be considered halal, such as ensuring that the materials used are from permissible sources and that there is no contamination from non-halal substances during the production process.

Bioplastics derived from starch, cellulose-based materials, chitosan derived from shrimp and crab shells, and bamboo fibres are some examples of halal biomaterials for food packaging. These materials are renewable, biodegradable, and compostable, making them eco-friendly and sustainable packaging options for food. In addition to meeting the requirements of Islamic law, packaging made from halal biomaterials is an environmentally responsible solution for the packaging industry. In the current Malaysian market, NLYTech Biotech located in Simpang Ampat, Pulau Pinang, and ADA Biotech based in Butterworth, Pulau Pinang are prominent manufacturers specializing in the production of halal-certified biomaterials for packaging applications. These manufacturers use a variety of halal-certified biomaterials, such as broken rice flour, tapioca starch, bamboo, natural food-grade colouring, and even used coffee grounds, to create environmentally friendly and halal-compliant packaging solutions. Their commitment to using permissible sources under Islamic law and meeting the stringent requirements for halal certification exemplifies the industry’s commitment to meeting the needs of Muslim consumers who seek environmentally friendly and ethical products.

Meanwhile, halal bioactive materials for intelligent food packaging refer to materials that comply with Islamic law and are utilised in the development of intelligent food packaging. These materials interact with the food product and provide information about the food’s freshness or safety, thereby preventing food spoilage and food waste caused by spoilage. Ultimately, the primary distinction between halal and non-halal intelligent packaging materials is that halal materials must be derived from Islamic law-compliant sources [71]. Another distinction is the manufacturing process. Halal bioactive materials must be produced in a halal-compliant manner, such as by avoiding cross-contamination with prohibited substances or by using equipment that has not been exposed to prohibited substances.

There is no discernible difference in terms of performance between halal and non-halal bioactive materials. Both can significantly improve the preservation and shelf life of food products. Examples of halal bioactive materials for intelligent food packaging indicators include pH indicators that change colour in response to changes in the acidity of the food product, oxygen indicators that change colour in response to changes in the oxygen levels inside the package, and microbial indicators that change colour in response to the growth of bacteria and other microorganisms in food products. Due to consumers’ growing concerns about the use of synthetic additives in food products, the food industry has increasingly turned to natural colourants as an alternative to synthetic colourants [72].

In the market of food colouring, natural pigments such as carmine, annatto, and curcumin are prevalent. The excellent tinctorial qualities of cochineal carmine, a dye produced by *Dactylopius coccus* insects, are highly prized. It is a high-demand functional dye due to its superior antioxidant activity, which is comparable to that of well-known antioxidants such as quercetin, ascorbic acid, and Trolox [73]. According to Islamic law, cochineal carmine is halal because the insects used to produce it are not killed or harmed during extraction, and an insect whose blood does not flow is deemed pure. The Malaysian Fatwa Committee and the Malaysian Food Regulations have permitted the use of cochineal dye because it is manufactured in accordance with Good Manufacturing Practises and does not pose a health risk [74]. There have been previous attempts to improve the mechanical and gas barrier properties of polyvinyl alcohol (PVA) films using a carminic acid-containing edible gel [75].

It should be emphasised that while the current halal certification of packaging materials available in the market is not specifically aimed at the development of bioactive materials for intelligent food packaging, it is still a critical step towards creating an industry-wide halal intelligent food packaging. This initiative demonstrates a strong commitment to advancing sustainable and halal-friendly practices in the packaging industry, and it sets the stage for future research and development of packaging materials that incorporate halal bioactive components for enhanced food safety and quality. By embracing these innovative solutions, the industry can continue to evolve and meet the growing demand for environmentally friendly and ethically sourced packaging solutions.

## 6. Recent Technologies

The changes in lifestyle, especially after the pandemic, have increased the demand for excellent packaging to preserve and maintain the good quality of food products. However, intelligent packaging systems are neither extensively nor globally spread in the whole market. This system is being practised by several countries that are fast-leading in technology, such as Japan, Canada, India, New Zealand, and the United Kingdom (Table 2) [76]. In Malaysia, intelligent packaging technology is still a new concept, but there is growing recognition of its potential benefits in terms of product safety, quality assurance, and supply chain management. Intelligent packaging has market potential in Malaysia, particularly in industries such as food and pharmaceuticals; however, due to several factors such as limited knowledge and skills, cost considerations, and a lack of regulatory frameworks, implementation and utilisation of this technology are still in their early stages.

**Figure 4:**
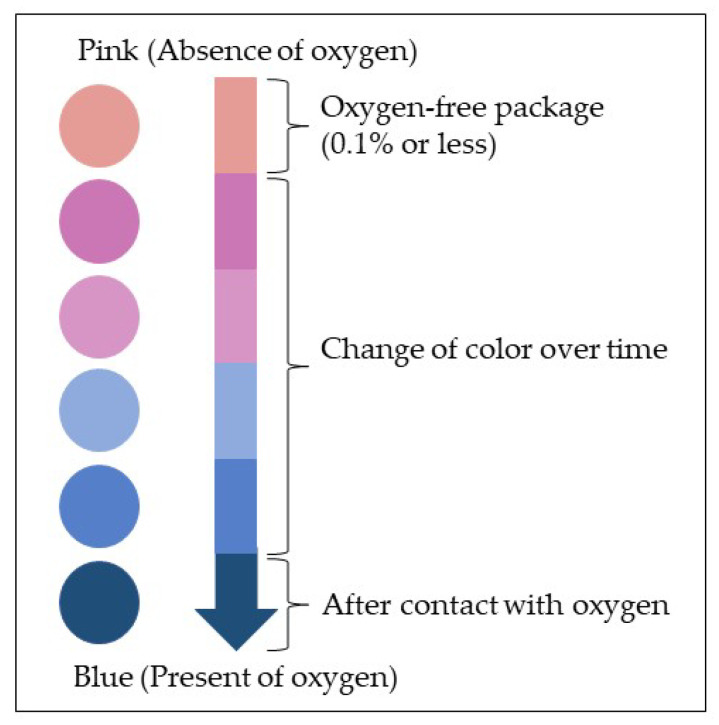
Schematic representation of leak indicators, reproduced from [78].

One of the factors why this technology is not widely used is the cost of production. The application of intelligent packaging is applied by considering the level it contributes towards increasing sales or reducing waste. In addition, it can be applied to perishable or sensitive products, such as poultry, meat, and seafood, given that the level of freshness and quality of these foodstuffs are not visually evident or known by the consumers [4]. Figure 4 shows an example of a freshness indicator, which is also known as a leak indicator, for intelligent food packaging. The colour changes from pink to blue by the level of oxygen present in the packaging within the time monitored [77,78].

Intelligent packaging technology has mainly been applied in the food industry, but it has the potential to benefit other fields, such as cosmetics and pharmaceuticals. The specific active and intelligent packaging technologies used will depend on the type of food product, with fresh fruits and vegetables potentially requiring packaging that controls humidity, while processed meats may require packaging that controls oxygen levels.

However, there are limitations to intelligent packaging technology, such as higher costs, complexity, and the limited availability of active materials. Additionally, specialised knowledge and expertise are needed due to the complexity and limited accessibility of the bioactive materials used in these technologies [79]. Despite the potential drawbacks, the advantages of extending shelf life and lowering food waste could outweigh the costs in the long run. Further research is needed to discover more bioactive materials that can be used in active materials in intelligent packaging systems.

The cost-effectiveness of active and intelligent packaging technology will depend on various factors, including the specific technology used, production scale, and resources and materials required. While the cost of intelligent packaging materials may be higher than conventional packaging, the extended shelf life and improved food safety can lead to reduced food waste, boost consumer confidence, and increase sales and market share [4]. Therefore, a cost–benefit analysis of each case is necessary to determine the cost-effectiveness of active and intelligent packaging technology.

## 7. Recommendation

**Figure 5:**
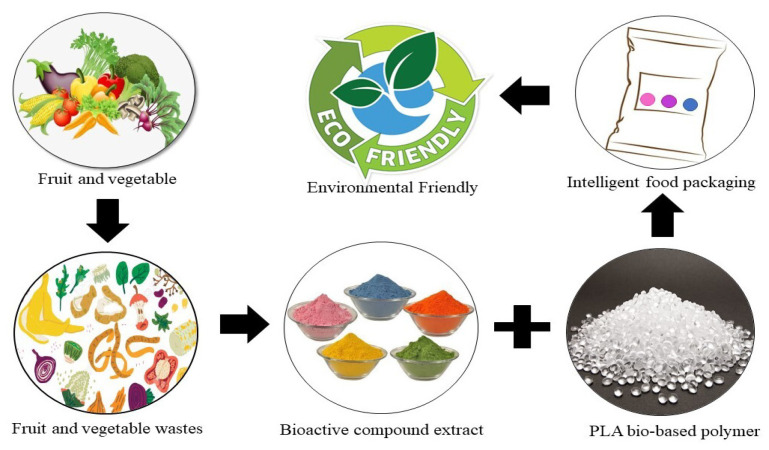
Production of intelligent food packaging using the PLA polymer incorporated with bioactive compounds extracted from fruit and vegetable wastes.

As a recommendation to enhance food quality and safety, the material and additives for intelligent packaging need to be considered because they indicate not only the condition of the surrounding environment but also the product itself. Additionally, the packaging should be well disposed of and remain environmentally friendly after it has been taken off the food. Thus, the incorporation of bio-based polymers with natural bioactive compounds as additives is an excellent solution to benefit the food industry as a whole. Figure 5 illustrates the example of intelligent food packaging development using the PLA polymer incorporated with bioactive compounds extracted from fruit and vegetable wastes. This technology not only improves food safety and quality but also preserves our precious earth.

As intelligent packaging technology continues to grow and expand globally, it is important to continue research and development to ensure its benefits are maximised. In particular, the use of halal bioactive materials derived from natural sources, such as plants and minerals, in intelligent packaging is gaining popularity due to its environmental friendliness and ethical production practices. To increase awareness and adoption of this technology among consumers and food manufacturers in Malaysia, several practical suggestions can be implemented. These include incorporating educational and informative content on product packaging, engaging in community outreach efforts, partnering with halal certification bodies, utilising social media platforms, offering incentives to businesses that adopt the technology, and collaborating with universities and research institutions to conduct further research and development. Raising awareness about halal bioactive materials as intelligent packaging technology is crucial to addressing critical issues such as food safety, sustainability, and market opportunities [80].

In addition, it is important to educate consumers about the benefits of this technology in the context of the risks and challenges that the food industry faces, including climate changes, pests and disease outbreaks, and other hazards such as COVID-19. By implementing these strategies, it is possible to improve the adoption and application of halal bioactive materials in intelligent packaging and contribute to a more sustainable and efficient food industry.

## 8. Conclusions

The research of active materials for intelligent packaging indicators is ongoing, intending to discover new and more effective compounds to incorporate into packaging as well as improve processing and manufacturing methods to create high-performance, environmentally friendly packaging. As halal goes beyond religious reasons to include ethical production, responsible consumption, and environmental protection, this paper provides state-of-the-art halal bioactive materials for intelligent packaging indicators that will meet the needs of the global food industry and consumers. Furthermore, global demand for halal-certified food products is rising, and the development of halal bioactive materials opens new opportunities for businesses to tap into this market.

## Figures and Tables

**Table 1:** Synthetic and natural active food packaging materials.

Compound	Food	Function	References
Roses	Shrimp	Freshness indicator	[47]
Sodium bicarbonate and citric acid	Chicken	Gas indicator	[48]
Red beet	Fish/shrimp	Freshness indicator	[49]
Betalain	Meat	Freshness indicator	[50]
Dragon fruit	Fish	Freshness indicator	[51]
Zinc oxides	Apple	Microbial indicator	[52]
Thyme herb	Bread	Freshness indicator	[53]
Nisin	Ham	Microbial indicator	[54]
Purple sweet potato	Meat	Time–temperature indicator	[55]
Pomegranate	Orange fruit	Microbial indicator	[56]
Roselle	Pork	Freshness indicator	[57]
Red cabbage	Pasteurised Milk	Time–temperature indicator	[58]
Eggplant	Milk	Freshness indicator	[59]
Clove essential oil and zinc oxide	Shrimp	Microbial indicator	[60]
Red cabbage	Milk	Freshness indicator	[61]
Jambolan fruit	Shrimp	Time–temperature indicator	[62]
Lactoferrin	Fresh sausages	Microbial indicator	[63]
Black rice bran	Pomfret/shrimp	Freshness indicator	[64]

**Table 2:** Intelligent packaging indicators are commercially available in the market [4,76].

Manufacturer	Product Name	Indicator	Information
Mitsubishi Gas Chemical Inc. (Tokyo, Japan)	Ageless Eye^TM^	Gas	Notifies the presence/absence of oxygen by changing the colour from pink to blue when the oxygen level > 0.5%.
Freshpoint Lab (Epping, VIC, Australia)	O_2_ Sense	Gas	Colour changes when a package is damaged, or when oxygen is detected inside the packaging.
Insignia Technologies Ltd. (Scotland, United Kingdom)	Novas	Gas	Specially made for plastic packaging and detects packaging degradation by changing colour.
Ripesense Limited (Tauranga, New Zealand)	RipeSense	Gas	Colour changes from red to yellow when the fruit ripens.
Vanprob (Maharashtra, India)	Food Fresh^TM^	Freshness	Colour changes when food is not fresh
DSM NV and Food Quality Sensor International (Lexington, MA, USA)	SensorQ^TM^	Freshness	Used for poultry/meat products to detect bacteria growth.
Toxin Alert (Etobicoke, ON, Canada)	SIRA	Freshness	Barcode will indicate the presence of bacteria
COX Technologies (Belmont, North Carolina)	Fresh Tag	Freshness	Colour changes to intense pink when fish/seafood releases odour.
Timestrip UK Ltd. (Cambridge, United Kingdom)	Timestrip	Time–temperature	Can monitor time and temperature from days to months depending on the types of products.
3M (St. Paul, MI, USA)	Monitormark^TM^	Time–temperature	Monitor the temperature during storage and transportation starting from −15 °C to 26 °C
EVIGENCE SENSORS (Hoboken, NJ, USA)	Evigence sensors	Time–temperature	A silver colour indicator changes to white by increasing temperature; monitoring ranges from hours to years; have a SMARTDOT^TM^ app for mobile usage.
Pymah Corp. (City of Los Angeles, CA, USA)	Cook-Chex	Time–temperature	A purple colour tag will turn green when exposed to pure steam atmosphere temperature.
Temptime Corp. (Morris Plains, NJ, USA)	Fresh-Check	Time–temperature/Freshness	Monitors food after purchasing in real-time. The circle darkens if the product is kept at an inappropriate temperature.

## Data Availability

No new data were created or analysed in this study. Data sharing is not applicable to this article.
